# Loss of functional connectivity in migration networks induces population decline in migratory birds

**DOI:** 10.1002/eap.1960

**Published:** 2019-07-22

**Authors:** Yanjie Xu, Yali Si, Yingying Wang, Yong Zhang, Herbert H. T. Prins, Lei Cao, Willem F. de Boer

**Affiliations:** ^1^ Department of Earth System Science Ministry of Education Key Laboratory for Earth System Modelling Tsinghua University Beijing China; ^2^ Resource Ecology Group Wageningen University and Research Wageningen The Netherlands; ^3^ College of Biology and the Environment Nanjing Forestry University Nanjing China; ^4^ State Key Laboratory of Urban and Regional Ecology Research Center for Eco‐Environmental Sciences Chinese Academy of Sciences Beijing China

**Keywords:** bird migration, habitat loss, life history, network robustness, population dynamics, species traits, wetland

## Abstract

Migratory birds rely on a habitat network along their migration routes by temporarily occupying stopover sites between breeding and non‐breeding grounds. Removal or degradation of stopover sites in a network might impede movement and thereby reduce migration success and survival. The extent to which the breakdown of migration networks, due to changes in land use, impacts the population sizes of migratory birds is poorly understood. We measured the functional connectivity of migration networks of waterfowl species that migrate over the East Asian‐Australasian Flyway from 1992 to 2015. We analysed the relationship between changes in non‐breeding population sizes and changes in functional connectivity, while taking into account other commonly considered species traits, using a phylogenetic linear mixed model. We found that population sizes significantly declined with a reduction in the functional connectivity of migration networks; no other variables were important. We conclude that the current decrease in functional connectivity, due to habitat loss and degradation in migration networks, can negatively and crucially impact population sizes of migratory birds. Our findings provide new insights into the underlying mechanisms that affect population trends of migratory birds under environmental changes. Establishment of international agreements leading to the creation of systematic conservation networks associated with migratory species’ distributions and stopover sites may safeguard migratory bird populations.

## Introduction

Recent trends in habitat loss and degradation strongly impact the survival and reproduction of wildlife species (Cushman 2006, Studds et al. 2017, Xu et al. 2017). Habitat loss and degradation reduce functional connectivity, i.e., the degree to which landscape elements promote animal movements within and between habitat patches (Taylor et al. 1993, Bélisle 2005, Saura and Rubio 2010). Functional connectivity links landscape features with species’ dispersal traits and is critical for understanding how the spatial distribution of suitable landscapes may influence populations of migratory species. The functional connectivity of migration networks is defined here as the degree to which the habitat configuration facilitates bird movements both within and between the breeding, non‐breeding, and stopover sites.

Many migratory birds rely on a network of habitat patches as they travel between breeding and non‐breeding areas. For example, many birds take advantage of multiple stopover sites during their migration for resting or refueling before migrating further and breeding (Arzel et al. 2006, Newton 2010, Si et al. 2018). Hence, connectivity among sites along migration flyways is essential for their survival and reproduction (Merken et al. 2015) and thereby can play a vital role in the population dynamics of these species. A stable network of stopover sites is an important component for maintaining stable or increasing populations of migratory species (Leito et al. 2015). In contrast, loss of habitat and loss of network connectivity may negatively impact migratory bird populations (Iwamura et al. 2013).

The degree to which losses in the functional connectivity of a migration network, as a consequence of habitat loss and degradation, contribute to population declines in migratory birds remains unknown. So far, the relationships between functional connectivity of migration networks and population trends have not been investigated empirically (Gilroy et al. 2016, Barshep et al. 2017, Studds et al. 2017). One reason for this is the challenge of quantifying the connectivity of habitat networks at a flyway scale over extended periods of time, though such quantification at these relatively large spatial and temporal scales is essential when the goal is to assess links between functional connectivity and population dynamics of migratory species.

We hypothesize that functional connectivity, as well as other previously studied predictors, together drive population trends of migratory birds, so that population sizes of migratory birds decrease with a decreasing functional connectivity of their migration networks. Species, such as those with longer migration distances (Morrison et al. 2013), with a smaller size of their non‐breeding ranges relative to breeding ranges (migratory dispersion; Gilroy et al. 2016), and with smaller breeding ranges (Murray et al. 2014), are more likely to experience population declines. Others, such as species with a relatively small body mass (de Boer et al. 2011), large clutch size (Jiguet et al. 2007), and short generation length (Murray et al. 2014), are less likely to decline.

To test this hypothesis, we measured the functional connectivity of migration networks of eight waterfowl species that winter in the Yangtze River Basin and migrate over the East Asian‐Australasian Flyway. We tested whether a decrease in functional connectivity was correlated with a decrease in non‐breeding population sizes of migratory waterfowl species from 2001 to 2014. Additionally, we included six common predictors for population dynamics: breeding range size, migratory dispersion, migration distance, body mass, generation length, and clutch size. The results can provide new insights into the underlying mechanisms that affect population trends of migratory species, and point out efficient conservation strategies for safeguarding the sustainability of migratory birds under observed land use changes.

## Methods

### Estimate bird population sizes

We selected eight waterfowl species that have a majority of their East Asian‐Australasian flyway population wintering in the Yangtze River Basin, China (Cao et al. 2010): Tundra Swan (*Cygnus columbianus*), Swan Goose (*Anser cygnoid*), Bean Goose (*Anser fabalis*), Greater White‐fronted Goose (*Anser albifrons*), Lesser White‐fronted Goose (*Anser erythropus*), Greylag Goose (*Anser anser*), Common Teal (*Anas crecca*), and Northern Pintail (*Anas acuta*). The flyway population trends for each of these species were estimated by Anatidae non‐breeding counts between 2001 and 2014 in the Yangtze River Basin (Zhang et al. 2015). The non‐breeding population sizes of each year were estimated by the sum of birds counted in all lakes in the Yangtze River Basin, and trends in population sizes were illustrated using a locally weighted scatterplot smoothing (LOWESS) method. Lakes without counting data in certain years were interpolated by means of nearest neighbors. The eight study species in a total of 24 lakes were observed annually during the 14‐yr study period during 2001–2014; thus, we included a total of 2,688 records in the analysis.

### Quantify functional connectivity of migration networks

**Figure 1:**
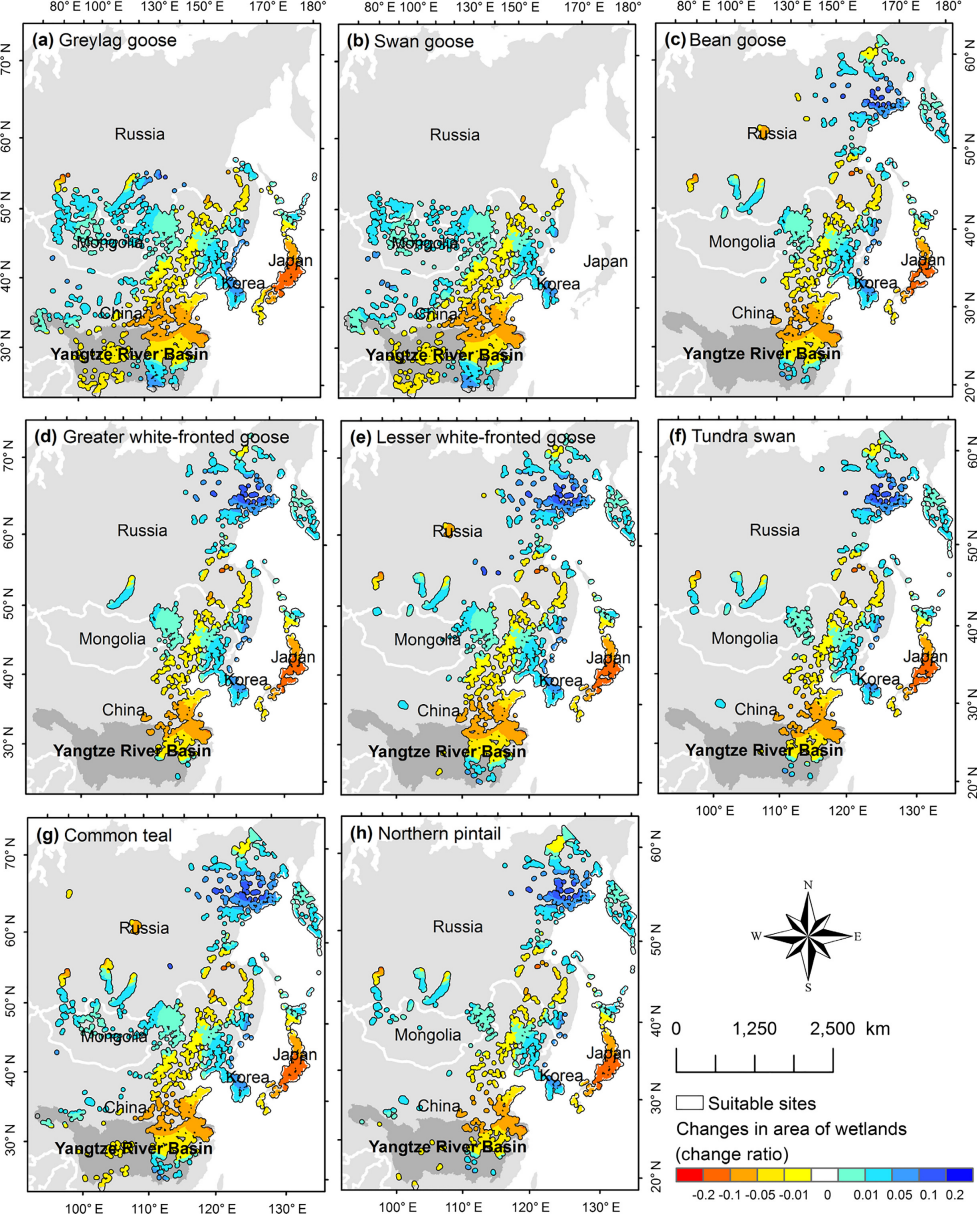
Patterns of habitat loss in the ranges of study species. The suitable sites in the East Asian‐Australasian Flyway for each study species and change ratios of the area of wetlands in the suitable sites during 1992–2012 were both analyzed in a previous study (Xu et al. 2019). Each connected wetland patch (within a distance of 32.5 km) in the suitable sites was defined as a node of the migration networks. Blue indicates the increase of wetland area and red indicates the decrease of it. The map was produced with ArcMap 10.2 (ESRI, Redlands, California, USA) under the cylindrical equal area projection.

For every year (1992–2015), we constructed migration networks of each of the study species, in which the “nodes” were the connected wetland patches in the suitable sites for each study species in the East Asian‐Australasian Flyway (Fig. 1; Xu et al. 2019). Distances between any two nodes were set at >32.5 km, which is the mean of maximum foraging flight distance of geese and ducks (Johnson et al. 2014). We defined the strength of node‐to‐node connections by the dispersal probability of the study species’ direct movement between two nodes (internode dispersal probabilities). Because a limited number of birds have been ringed or fitted with telemetry equipment, we could not rely on direct calculations of dispersal probabilities between sites. Therefore, we used an indirect method, a decreasing exponential function (Keitt et al. 1997), to quantify internode dispersal probabilities

(1) $$P_{\mathrm{ij}}=e^{-kd_{\mathrm{ij}}}$$

*P*_*ij*_ is the dispersal probability between node *i* and *j*, and *d*_*ij*_ is the closest distance between habitat patch *i* and *j*,* k* is a constant defined so that dispersal probability is 50% when *d*_*ij*_ equals the median of the migration lags of the study species, i.e., distances between consecutive sites used by migratory birds (Appendix S1: Table S1).

To facilitate comparison of functional connectivity of migration networks between years and species, we measured the functional connectivity of a migration network through an index of “equivalent connected area” (Saura and Pascual‐Hortal 2007, Saura et al. 2014) in square kilometers using a directed graph theory algorithm (Saura et al. 2014). Equivalent connected area is the size of a single patch providing the same level of connectivity as the calculated migration network (Eq. (2)). A larger equivalent connected area means a better‐connected migration network:

(2) $$\text{ECA}=\sqrt{\sum_{i=1}^{n}\sum_{j=1}^{n}a_{i}a_{j}P_{\mathrm{ij}}^{*}}$$

where *a*_*i*_ and *a*_*j*_ represent the area (km^2^) of habitat sites (i.e., nodes) *i* and *j*. *P*_*ij*_ * is the maximum product probability, i.e., the “best” paths with one or more steps between nodes *i* and *j*. When *P*_*ij*_ * =* P*_*ij*_, nodes *i* and *j* are close enough for individuals to move directly between them. When *P*_*ij*_ * > P_*ij*_ *,* the “best” path consists of several steps within the network and involves stepping stones in between nodes *i* and *j*. The functional connectivity values were decomposed (contribution, %) into intra, direct, and step fractions, showing the contribution (%) of within‐patch connectivity, direct connections, and use of stepping stones between source and destination patches, respectively (mathematical details are available in Saura et al. 2014). Specifically, intrapatch connectivity is the fraction corresponding to the area of reachable habitat within sites used by migratory birds. Direct connectivity is the amount of intersite connectivity if stepping stones are not used in the movements of birds among sites. Step connectivity is the increase in the amount of connectivity by having higher probabilities of movement between sites due to the use of existing stepping stones. We ran the analyses in R 3.3.1 (R Development Core Team 2018) with Conefor 1.1.6 for directed networks (Saura and Torne 2009).

### Additional predictors for bird population changes

We included six species traits as potential predictors in the analyses, i.e., body mass (g), breeding range size (km^2^), clutch size (the average number of eggs laid; *N* per female), generation length (yr), migratory dispersion, and migration distance (km). Migratory dispersion is the size of species non‐breeding range relative to that of breeding range (Gilroy et al. 2016). We calculated migratory dispersion, breeding range size, and migration distance on the basis of species’ distribution maps (Birdlife International and NatureServe 2015). We measured both migratory dispersion and breeding range size using a cylindrical equal area projection. Breeding range size was the total area of breeding ranges of a study species. Migratory dispersion was measured by the difference between the log‐transformed area of the non‐breeding area and that of the breeding ranges divided by log‐transformed area of the breeding ranges of a study species (Gilroy et al. 2016). We measured migration distance as the distance between the centroids of breeding and non‐breeding ranges of a study species using an azimuthal equidistant projection. Body mass and clutch size of the study species were obtained from the amniote life‐history database (Myhrvold et al. 2015). Generation lengths were obtained from the IUCN Red List of Threatened Species (Birdlife International 2016).

### Statistical analysis

We tested for differences in functional connectivity of migration networks among the different study species using a one‐way ANOVA test followed by Tukey's post hoc tests. Residuals were normally distributed (Kolmogorov‐Smirnov test, *P* > 0.05).

Phylogenetic non‐independence among species can bias results, and we therefore implemented a phylogenetically corrected model, i.e., a multi‐variable phylogenetic linear mixed model (PLMM; Pearse et al. 2015), to test for the effects of functional connectivity of migration networks and other additional predictors on the population changes of the study species. Data of all study species in each of the survey years (14 yr; 2001–2014) were included in the PLMM. We fitted random effect terms that account for phylogenetic co‐variance (Ives and Helmus 2011), bird species, and year of observation. We acquired a subset tree of the study species based on the Ericson backbone (Ericson et al. 2006) from BirdTree.org (Jetz et al. 2012). The dependent variable was the population change ratio (PCR; Eq. (3)). PCR was calculated as the difference between the population size in a given year *i* (*P*_*i*_) and the population size of a starting year (*P*_2001_) divided by the population size of the starting year:

(3) $$\text{PCR}=\frac{P_{i}-P_{2001}}{P_{2001}}$$

Independent variables (described above) included body mass, breeding range size, migratory dispersion, clutch size, generation length, migration distance, and changes in functional connectivity of migration networks of each species. Changes in functional connectivity (CFC) were calculated as the difference in functional connectivity of migration networks between a given year *i* (FC_*i*_) and the starting year (FC_2001_):

(4) $$\text{CFC}={\text{FC}}_{i}-{\text{FC}}_{2001}$$

We removed one independent variable with a variance inflation factor larger than 10 (generation length) to reduce the effect of multicollinearity. All factors were scaled to facilitate comparison of the contributions to the prediction. Interaction terms were not fitted in the model due to limited sample sizes. The best model with the smallest Bayesian Information Criterion (BIC) was selected by a backward elimination procedure (Burnham and Anderson 2003). To account for lag effects of changes in functional connectivity of migration networks on changes in population sizes, we fitted the PLMMs with different lag periods (i.e., 1–7 yr; Table 1; Appendix S1: Fig. S1). In these lag models (Table 1), the change in functional connectivity CFC (Lag *n*) was calculated as the difference in functional connectivity (FC) between a number of years (i.e., the number of lags: *n*) before the bird survey year (*i*) and year 1992, the starting year of the functional connectivity measurement:

**Table 1:** A test for lag effects of changes in functional connectivity on changes in population sizes for eight waterfowl species, showing the performance of the best models and regression coefficients for predictors included in these models

Model	Coefficient	Standard error	*z*	*P*
Lag 1 (*N* = 112, BIC = 319.9)				
(Intercept)	0.175	0.253	0.691	0.490
Changes in functional connectivity	0.235	0.100	2.359	0.018*
Lag 2 (*N* = 112, BIC = 319.4)				
(Intercept)	0.175	0.253	0.693	0.488
Changes in functional connectivity	0.245	0.098	2.494	0.013*
Lag 3 (*N* = 112, BIC = 318.9)				
(Intercept)	0.175	0.250	0.699	0.484
Changes in functional connectivity	0.254	0.095	2.674	0.007*
Lag 4 (*N* = 112, BIC = 318.8)				
(Intercept)	0.175	0.249	0.703	0.482
Changes in functional connectivity	0.257	0.094	2.749	0.006*
Lag 5 (*N* = 112, BIC = 319.1)				
(Intercept)	0.176	0.249	0.705	0.481
Changes in functional connectivity	0.252	0.094	2.668	0.008*
Lag 6 (*N* = 112, BIC = 319.2)				
(Intercept)	0.177	0.250	0.708	0.479
Changes in functional connectivity	0.249	0.093	2.677	0.007*
Lag 7 (*N* = 112, BIC = 321.9)				
(Intercept)	0.173	0.252	0.688	0.492
Changes in functional connectivity	0.185	0.101	1.824	0.07

*Notes:* The lag periods for measuring changes in functional connectivity are in units of years, i.e., in the model Lag 1, changes in functional connectivity were measured by the change 1 yr before the corresponding population count. The number of lags represents the changes in functional connectivity over the number of years.

* The estimated regression coefficient was significant at *P* ≤ 0.05.

(5) $$\text{CFC}\;\left(\text{Lag}\;n\right)={\text{FC}}_{i-n}-{\text{FC}}_{1992}$$

## Results

### Differences among species

**Figure 2:**
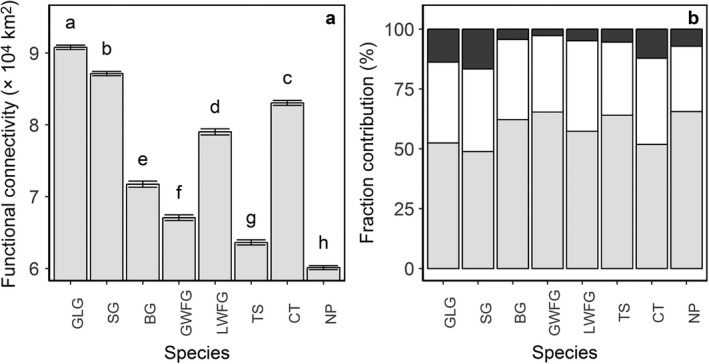
Functional connectivity of migration networks among bird species. (a) The eight study species include the Greylag Goose (GLG) and Swan Goose (SG), Bean Goose (BG), Greater White‐fronted Goose (GWFG), Lesser White‐fronted Goose (LWFG), Tundra Swan (TS), Common Teal (CT), and Northern Pintail (NP). The equivalent connected area (km^2^ × 10,000) averaged over 1992–2015 is presented per species (mean ± SE). Letters represent group differences as identified by Tukey's multiple comparison test (*P *≤* *0.05). (b) Contribution of fractions show intrapatch (light gray), direct (white), and step (dark gray) connections in percentages of functional connectivity.

Average functional connectivity of migration networks over the period 1992–2015 significantly differed among the eight study species (Fig. 2a; one‐way ANOVA, *F*_7, 184_ = 23,667, *P *<* *0.001). Among these species, the migration network of the Northern Pintail (*Anas acuta*) was the least connected with an equivalent connected area of 601,000 km^2^. The Greylag Goose (*Anser anser*, equivalent connected area = 908,000 km^2^), Swan Goose (*Anser cygnoid*, equivalent connected area = 871,000 km^2^), and Common Teal (*Anas crecca*, equivalent connected area = 831,000 km^2^) had the largest connected migration networks (Fig. 2a).

Intrapatch connectivity contributed more than one‐half the functional connectivity of migration networks for all species. Step connections contributed least, especially for the swan and goose species with major breeding grounds in Russia (i.e., 2% for Greater White‐fronted Goose (*Anser albifrons*), 4% for Bean Goose (*Anser fabalis*), 5% for Lesser White‐fronted Goose (*Anser erythropus*), and 5% for Tundra Swan (*Cygnus columbianus*), Fig. 2b).

### Changes over time

**Figure 3:**
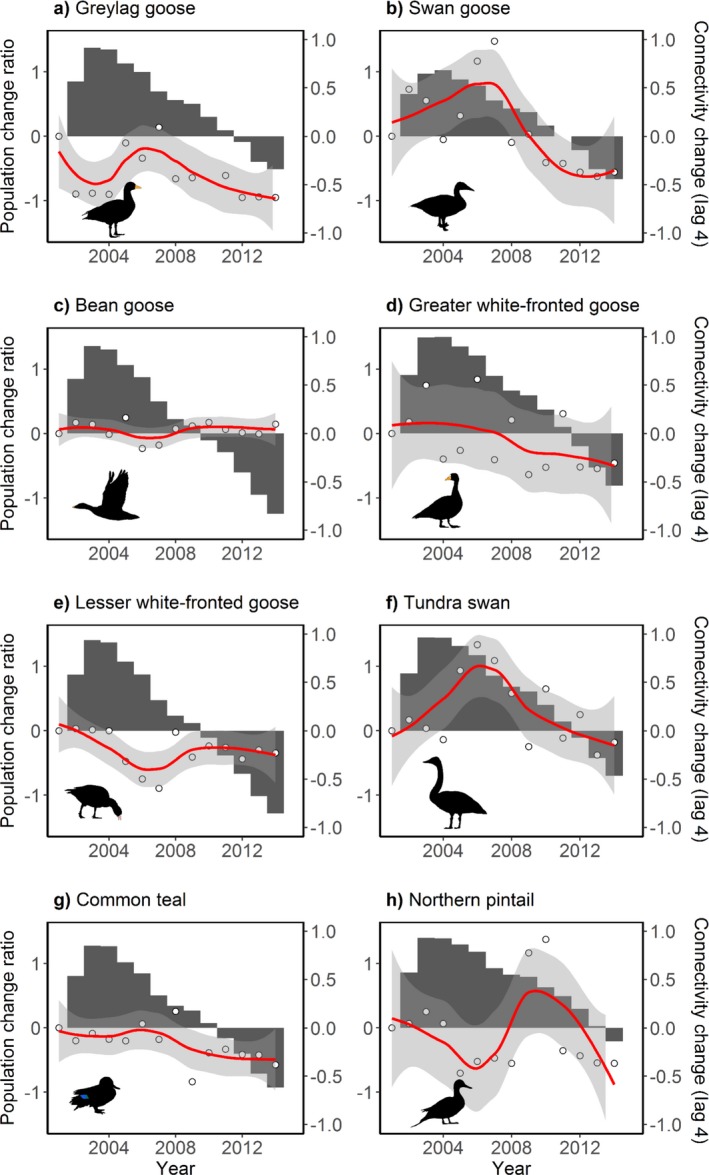
Changes in bird population sizes and in functional connectivity of migration networks. Population change ratio is the difference between the population size in a given year and the population size in 2001 divided by the population size in 2001. Population change ratios are displayed with dots and their trends are represented by smoothed red lines, using a locally weighted scatterplot smoothing method. Connectivity change (gray bars) with a 4‐yr lag is presented as the difference in the equivalent connected area (km^2^) of the migration network between 4 yr before a given year and year 1992. To facilitate comparison between species, connectivity change (indicated by dark grey bars) is standardized by being divided by the maximum connectivity change. A 4‐yr lag is displayed because the population changes are best explained by the changes in functional connectivity 4 yr before the survey year (Table 1).

During 2001–2014, the estimated non‐breeding population sizes of the Greylag Goose, Swan Goose, Tundra Swan, Common Teal, and Northern Pintail first increased and then decreased; population sizes of the Bean Goose, Greater White‐fronted Goose, and Lesser White‐fronted Goose first decreased and then either stabilized or slightly increased over the survey period (Fig. 3). Generally, the study species showed a decreasing trend, and at the meantime, the functional connectivity of migration networks of all the eight species consistently and continuously declined since 2001. However, before 2001 (i.e., 1994–1999; periods differ among species), there was an increase in the functional connectivity.

### Loss of functional connectivity affects bird population dynamics

**Figure 4:**
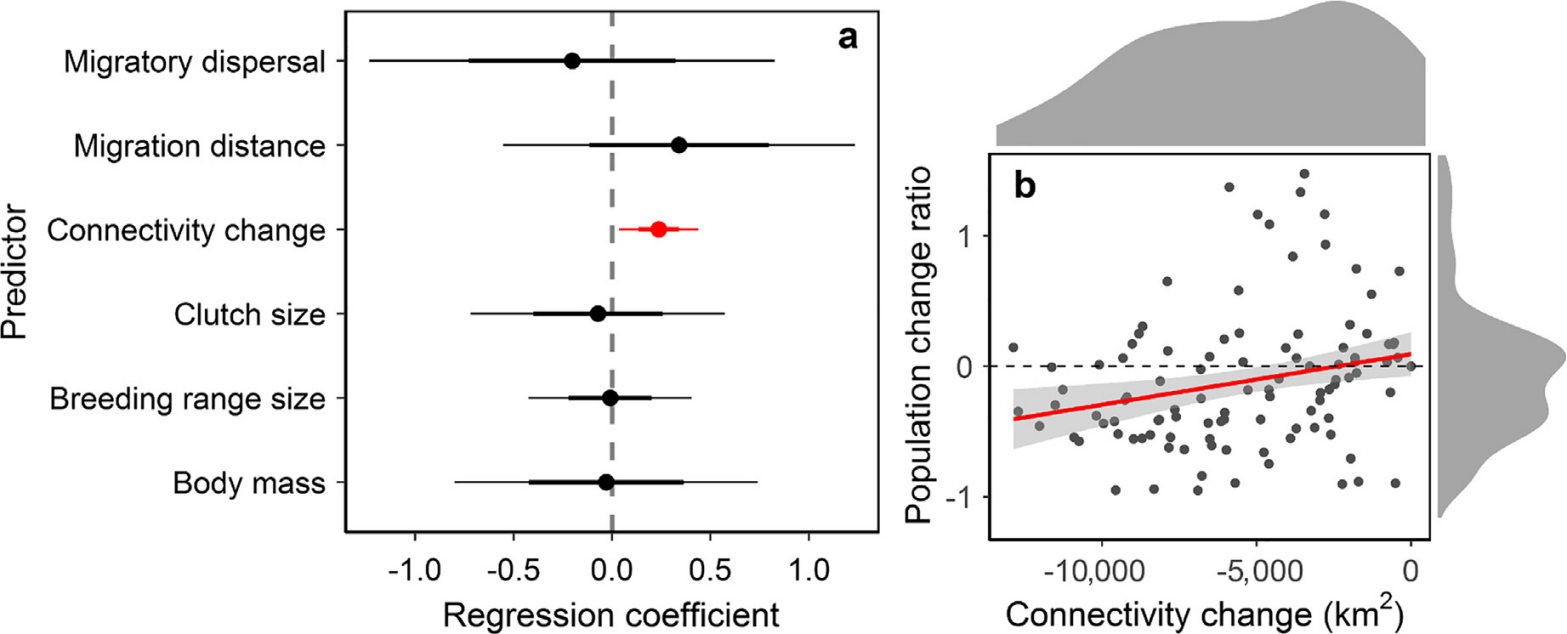
Changes in functional connectivity of migration networks is the only significant predictor for population declines of migratory birds. (a) Estimated coefficients ±95% (thin lines) and 68% confidence interval (thick lines) of predictors for population change ratio using a full model of multivariable phylogenetic linear mixed model. The significant predictor is in red. (b) The effect of changes in functional connectivity on population change ratios of the eight study species. We measured population change ratios using 2001 as the baseline year, the first year of the bird survey. The relationship between population change ratio and changes in functional connectivity were represented by a fitted line (red line) with 95% confidence intervals (gray area). When functional connectivity declines, populations decline. Density plots on top and right show distributions of connectivity change and population change ratio, respectively.

Among the seven analysed factors, generation length was collinear with other predictors (variance inflation factor >10), and was excluded from the PLMM. The population change ratio was significantly and positively related to changes in functional connectivity of migration networks (*N *=* *112, regression coefficient = 0.24, 95% confidence interval = 0.04–0.44, *P *=* *0.02; Fig. 4a; Table 2). No other variables showed a significant effect, and changes in functional connectivity was the only factor included in the best model (Table 2). When the functional connectivity declined, bird populations declined (Fig. 4b). There was a lag effect of functional connectivity on population changes of migratory birds, with a strongest effect of a 4‐yr lag on the population change ratio (*N *=* *112, regression coefficient = 0.26, 95% confidence interval = 0.07–0.44, *P *=* *0.01; Table 1; Appendix S1: Fig. S1).

**Table 2:** Results of the phylogenetic linear mixed model of species traits on changes in population sizes for eight waterfowl species, showing the performance of the models and regression coefficients for predictors included in these models

Model	Coefficient	Standard error	*z*	*P*
Full model (*N* = 112, BIC = 324.2)				
(Intercept)	0.199	0.450	0.442	0.658
Body mass	−0.030	0.393	−0.076	0.940
Breeding range size	−0.010	0.211	−0.048	0.962
Changes in functional connectivity	0.238	0.103	2.308	0.021*
Clutch size	−0.072	0.329	−0.219	0.827
Migration distance	0.341	0.456	0.748	0.455
Migratory dispersal	−0.203	0.525	−0.386	0.699
Best model (*N* = 112, BIC = 320.7)				
(Intercept)	0.169	0.256	0.661	0.508
Changes in functional connectivity	0.216	0.100	2.163	0.031*

*Note:* The full model and the best models with a smallest Bayesian information criterion (BIC) are listed.

* The estimated regression coefficient was significant at *P* ≤ 0.05.

## Discussion

We found that loss of functional connectivity in migration networks is a crucial predictor for population declines in migratory birds. Changes in functional connectivity was the only significant factor in the model predicting population changes, outperforming other previously used predictors of population decline and local extinction of migratory birds. Although migratory birds have high flexibility in distribution and migration, their populations declined with a decrease in functional connectivity. Moreover, population sizes responded immediately to connectivity loss, however, when a lag effect was taken into account in the analysis, the impact of changes in functional connectivity on population sizes became even stronger (Table 1; Appendix S1: Fig. S1). The impact of decreasing functional connectivity on population sizes can last long, and was largest using a 4‐yr lag effect.

With the loss of connectivity in their migration network, migratory birds must either adapt to suboptimal resources or accept suboptimal strategies (Weber et al. 1999, Schmaljohann and Both 2017, Si et al. 2018), e.g., longer non‐stop flights and/or suboptimal arrival, departure, and residence times. These adjustments could lead to increasing costs of migration and decreasing efficiency in energy refueling (Goymann et al. 2010); consequently, mortality during migration could increase and breeding success could decline. Carryover effects (Norris et al. 2004) could result in conditions becoming tougher over time for those species that successively lose habitat area along their migration routes. Ultimately, this loss of functional connectivity in migration networks could make it difficult to replenish energy stores during migration and maintain optimal body reserves for reproduction (Norris and Taylor 2006).

A well‐connected network facilitates animal movements and subsequent survival and viability (Crooks and Sanjayan 2006), especially for migratory birds, which pass through long and narrow geographic ranges twice a year. As for the study species, Greylag Goose and Swan Goose with relatively wider and shorter migration extents, have relatively well‐connected migration networks than the other species (e.g., Northern Pintail, Tundra Swan, and Greater White‐fronted Goose). A well‐connected migration network provides sufficient alternative routes for migrants, and not only promotes migratory movements, but also provides more possibilities for range shifts to cope with area‐specific environmental changes. Migratory species that rely on a migration network that is continuously losing connectivity are more likely to experience a population decline. Hence, connectivity of migration networks is an essential element for habitat change analysis for migratory species. Establishment of systematic conservation networks across species migration extents based on international agreements, e.g., the Ramsar Convention on Wetlands of International Importance and the European Union's Biodiversity Strategy 2020, should be comprehensively considered for biodiversity conservation planning.

Stepping stones are essential during seasonal migration. Even during Sahara crossing, migratory birds take short diurnal stopovers in resource‐poor desserts (Schmaljohann et al. 2007) as stepping stones, and birds crossing the Himalayas do the same (Prins and Namgail 2017). However, the step connections in the studied migration networks contributed least to the functional connectivity, which indicated a warning signal that these studied waterfowl species lack stepping stones to facilitate movements between sites. Successive loss of crucial stepping stones in migration routes can lead to the collapse of migration networks, so that migratory movements between breeding and non‐breeding grounds could be completely impeded (Shimazaki et al. 2004). Thus, it is necessary to put an emphasis on protecting critical sites used as stepping stones in migration to enhance the connectivity between isolated sites.

Human‐induced and climate‐driven changes to natural land cover can have large impacts on the connectivity of animal movement networks. Composition and structure of landscape mosaics can explain large‐scale species distribution and richness patterns especially for birds (Xu et al. 2014, Zhang et al. 2018). As indicated by our study, the functional connectivity of migration networks for species in the East Asian‐Australasian Flyway was continuously decreasing from the 2000s. Wetlands are one of the world's mostly threatened habitat types, under influence of climate change and human‐induced habitat destruction (Millennium Ecosystem Assessment 2005, Silva et al. 2007). Wetland loss can isolate and eliminate habitat sites in migration networks, thereby reducing network connectivity. Under high human–bird conflicts of East Asian flyways (Si et al. 2018, 2015), wetlands in this region are widely destroyed by human activities.

Migratory birds that rely the most on degraded stopover sites experienced the largest population decline (Studds et al. 2017) and habitat conditions during migration can influence bird survival (Hewson et al. 2016). Decreasing wetland area and food availability (e.g., via loss of grasslands) can lead to staging sites no longer being utilized by migrants (Verkuil et al. 2012, Zou et al. 2016). The vulnerability of a migratory species that uses a number of wetland sites increases even when only part of the network is negatively affected (e.g., by sea‐level rise or other human‐induced changes (Iwamura et al. 2013). Upon the degradation or loss in individual sites, the chance of a breakdown in both direct and indirect connections between sites increases. It is therefore essential to maintain well‐connected habitat networks by either expanding the area of (protected) sites or by adding new sites to existing networks, to increase the resilience of migrants to environmental change.

Species traits are often associated with population dynamics, as species that vary in traits respond differently to environmental changes (Gaston and Blackburn 1995, Pacifici et al. 2017). Individuals of large‐bodied species might be able to adapt to environmental changes more easily (Pacifici et al. 2017), as they are physiologically more resistant to environmental changes. Thus, species with larger body mass may have an advantage when environmental conditions deteriorate (Pacifici et al. 2017). The connectivity of the studied migration networks decreased continuously over the past 15 yr, under influence of deteriorating environmental conditions, such as habitat loss and fragmentation. However, the expectation that larger species were less affected by the decrease in function connectivity of their migration network could not be confirmed in our study, as the decline in population was similar among species. This is in agreement with, for example, the findings of Jiguet et al. (2007) who also found no relationships between the species body mass and their population trends. The extent to which the species’ life‐history traits affect their population dynamics is dependent on the geographical ranges and orders of species studied (Fritz et al. 2009). We fitted species as a random factor in the PLMM; the signal of the species traits may therefore be weakened. Our results indicate that a loss of functional connectivity of their migration network is a novel and crucial predictor for population declines of migratory birds. Further studies about population declines of migratory birds should take this factor into account. However, other species traits may also be important factors triggering population dynamics of migratory birds. This study included waterfowl species in the East Asian‐Australasian flyway, so future researches investigating whether our findings can be applied to a broader geographical or species range will be valuable.

## Data Availability

Data are available on the Dryad Digital Repository: https://doi.org/10.5061/dryad.r901kb6
