# Supplementary material for: Loss of functional connectivity in migration networks induces population decline in migratory birds
Source: Ecol Appl. 2019 Jul 22;29(7):e01960. doi: 10.1002/eap.1960 (PMC6852588; doi:10.1002/eap.1960)
Supplement: Supplementary file 1 [file EAP-29-na-s001.pdf]

Supporting Information. Yanjie Xu, Yali Si, Yingying Wang, Yong Zhang, Herbert H. T. Prins, Lei Cao, Willem F. de Boer. 2019. Loss of functional connectivity in migration networks induces population decline in migratory birds. *Ecological Applications*.

APPROVED

Appendix S1. Supplementary figure and table.

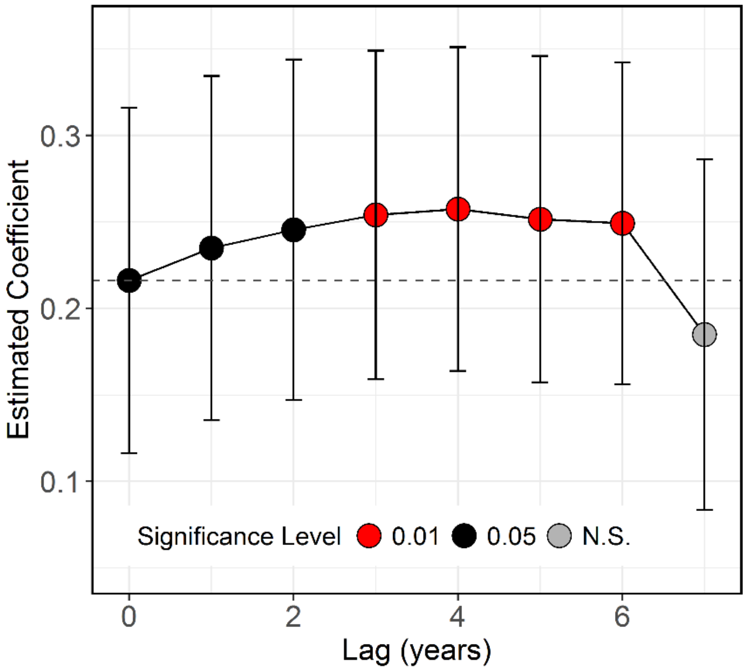

Figure S1. Effect of lag period on the relationship between functional connectivity of migration networks and changes in population sizes of migratory birds. Functional connectivity was calculated for different lag periods (years, x-axis). Estimated coefficients (± standard error) of the correlation between changes in functional connectivity and population sizes were obtained from multi-variable Phylogenetic Linear Mixed Models. The default model is with 0 lag and the dashed line represent the estimated coefficient from the default model to facilitate comparison between models. N.S. = not significant.

Table S1. Migration step length, i.e., distances between consecutive sites from tracking studies. 'Species group' stands for the category of species (geese, swans, or ducks), and major breeding grounds, i.e., border area between Mongolia, China, and Russia (geese 1) or Russia (geese 2). Common name and scientific name of focal species. Migration step length is the median distance (in km) of the migration steps between consecutive sites for a study species, as published in previous studies. For species where we could not find information on the median distance between steps in their migration network we took the value as published for the "indicator species".

Species group	Common name of study species	Scientific name	Indicator species	Migration step length (km)
Geese 1	Greylag Goose	*Anser anser*	*Anser cygnoides*	130 (Batbayar et al. 2013)
	Swan Goose	*Anser cygnoides*	*Anser cygnoides*	130 (Batbayar et al. 2013)
Geese 2	Bean Goose	*Anser fabalis*	*Anser albifrons*	550 (Kölzsch et al. 2016)
	Greater White-fronted Goose	*Anser albifrons*	*Anser albifrons*	550 (Kölzsch et al. 2016)
	Lesser White-fronted Goose	*Anser erythropus*	*Anser albifrons*	550 (Kölzsch et al. 2016)
Swans	Tundra Swan	*Cygnus columbianus*	*Cygnus columbianus*	571 (Beekman et al. 2002)
Ducks	Common Teal	*Anas crecca*	*Anas acuta*	258 (Miller et al. 2005)
	Northern Pintail	*Anas acuta*	*Anas acuta*	258 (Miller et al. 2005)

References

Batbayar, N., J. Y. Takekawa, S. H. Newman, D. J. Prosser, T. Natsagdorj, and X. Xiao. 2013. Migration strategies of Swan Geese *Anser cygnoides* from northeast Mongolia. Wildfowl **61**:90-109.

Beekman, J. H., B. A. Nolet, and M. Klaassen. 2002. Skipping swans: fuelling rates and wind conditions determine differential use of migratory stopover sites of Bewick's Swans *Cygnus bewickii*. Ardea **90**:437-460.

Kölzsch, A., G. J. Müskens, H. Kruckenberg, P. Glazov, R. Weinzierl, B. A. Nolet, and M. Wikelski. 2016. Towards a new understanding of migration timing: slower spring than autumn migration in geese reflects different decision rules for stopover use and departure. Oikos **125**:1496-1507.

Miller, M. R., J. Y. Takekawa, J. P. Fleskes, D. L. Orthmeyer, M. L. Casazza, D. A. Haukos, and W. M. Perry. 2005. Flight speeds of northern pintails during migration determined using satellite telemetry. The Wilson Bulletin **117**:364-374.
